# Ricci-flow based conformal mapping of the proximal femur to identify exercise loading effects

**DOI:** 10.1038/s41598-018-23248-y

**Published:** 2018-03-19

**Authors:** Nathaniel Narra, Shinya Abe, Vassil Dimitrov, Riku Nikander, Reijo Kouhia, Harri Sievänen, Jari Hyttinen

**Affiliations:** 1 BioMediTech Institute and Faculty of Biomedical Sciences and Engineering, Tampere University of Technology, Tampere, Finland; 2 Laboratory of Civil Engineering, Tampere University of Technology, Tampere, Finland; 3 Department of Electrical and Computer Engineering, University of Calgary, Calgary, Canada; 4 Department of Health Sciences, University of Jyväskylä, Jyväskylä, Finland; 5 GeroCenter Foundation for Aging Research and Development, Jyväskylä, Finland; 6 Jyväskylä Central Hospital, Jyväskylä, Finland; 7 The UKK Institute for Health Promotion Research, Tampere, Finland; 8 Geometric Energy Corporation, Calgary, Canada

## Abstract

The causal relationship between habitual loading and adaptive response in bone morphology is commonly explored by analysing the spatial distribution of mechanically relevant features. In this study, 3D distribution of features in the proximal femur of 91 female athletes (5 exercise loading groups representing habitual loading) is contrasted with 20 controls. A femur specific Ricci-flow based conformal mapping procedure was developed for establishing correspondence among the periosteal surfaces. The procedure leverages the invariance of the conformal mapping method to isometric shape differences to align surfaces in the 2D parametric domain, to produce dense correspondences across an isotopological set of surfaces. This is implemented through a multi-parametrisation approach to detect surface features and to overcome the issue of inconsistency in the anatomical extent present in the data. Subsequently, the group-wise distribution of two mechanically relevant features was studied – cortical thickness and surface principal strains (simulation results of a sideways fall). Statistical inferences over the surfaces were made by contrasting the athlete groups with the controls through statistical parametric mapping. With the aid of group-wise and composite-group maps, proximal femur regions affected by specific loading groups were identified with a high degree of spatial localisation.

## Introduction

Bone is an adaptive hard tissue which, among its other characteristics, is designed to be robust against physiological loads. The position of the bone and its function within the skeletal frame limits its range of motion through linkages between bones, joints and muscle attachments. This results in a constrained envelope of loading directions that a particular load bearing bone can experience during common habitual movements. In humans, the femur is indispensable for locomotion and load-bearing functions within a large range of motion under potentially high load magnitudes and impacts. The ability of the femur to withstand these loads is achieved through adaptive processes that modulate its morphology and composition. The cortical geometry at the proximal femur consequently reflects the most robust construction adapted for the specific loading it habitually experiences (e.g., locomotion and physical activity). Thus, consistent loading in specific directions, such as physical training of athletes over a long period of time, can induce corresponding local adaptations in the cortical geometry^1,2^. While loading stimuli have the most pronounced effect in adolescence^3^, their effectiveness in stimulating an adaptation decreases after bone reaches maturity^4^. However, it is known that the exercise-induced bone thickening during growth occurs through new bone formation on the periosteal bone surface, while age-related bone loss takes place at the endocortical bone surface^5^. Thus, beneficial geometric adaptations accrued during adolescence and young adulthood may have lasting benefits in senescence despite associated bone loss^6^. This was observed in retired (>60 years) ice-hockey and soccer players as reduced fracture risk, in comparison to age matched controls, despite some loss of exercise-induced gain in bone^7^. Moreover, studies have shown that beneficial bone adaptation can be observed through exercise even in older people^8^ and can help in arresting the attenuation of bone strength due to age-related bone loss^9^. Studying this cause-effect relationship between physical loading and regional adaptation is an area of active pursuit. Understanding the mechanisms that modulate specific adaptations at bone regions susceptible to fractures can be used as paradigms for preventing age-related degradation of bone robusticity, or alternatively, increased bone fragility. Also, establishing morphological features that result from specific loading patterns may help in recreating activity of past populations in the field of archaeological anthropology^10^.

Studies exploring the spatial heterogeneity in the adaptive response to physical loading have traditionally analysed bone cross-sections with respect to mechanically relevant (morphometric) features. To increase the detectability of this heterogeneity it is necessary to increase the spatial resolution of analyses. Within femoral neck cross-sections this has been achieved by partitioning into quadrants^1^, octants or higher angular divisions^2,11,12^ defined with respect to anatomical directions (e.g. superior, posterior, anterior). Access to 3D tomographic data has extended the potential for such detailed analyses. Concepts of computational anatomy^13^ and statistical parametric mapping^14^ make it possible to investigate the distribution of relevant parameters over 3D regions^15–17^ and assess their statistical significance. In large sample studies, establishing anatomical correspondence across samples is a prerequisite. Often noise and varying, or even incomplete, anatomical extents in the data can compromise the consistency in the shape registration process. Thus, methods that can provide computationally reliable registration across multiple surface instances can expand the tool-set beyond the commonly used iterative closest point (ICP) based methods^18^.

This study introduces a novel application of the conformal mapping method for establishing correspondence between proximal femur instances. The method treats the surface as a differentiable manifold and converts the 3D registration problem into 2D. This dimension reduction is achieved by a parametrisation procedure where the surface is conformally mapped to the planar domain using discrete surface Ricci-flow^19,20^ implemented in MATLAB (Release 2016b, The MathWorks, Inc., Natick, Massachusetts, United States). The advantages of this method in shape registration and indexing^21^ have been exhibited for anatomical objects such as brain^22^ and colon^23^. As a supporting case study, this registration approach was developed into a procedural chain to analyse the spatial distribution of geometric adaptation in the proximal femur in response to long-term habitual physical activity. The present dataset consists of young adult female athletes whose consistent and vigorous training regimen represented habitual activity. Geometric morphology was quantified in terms of the spatial distribution of cortical thickness. Adaptation was consequently defined spatially as regions exhibiting statistically significant differences from the controls. Adaptation can also be studied in terms of behaviour under specific mechanical loading. The recent finite element (FE) study conducted by Abe and colleagues simulated a ‘supra-physiological’ loading caused by sideways falling, to assess fracture risk of proximal femur^24^. Utilizing the results of this simulation, we illustrated the influence of adapted geometry in lowering fall-induced stresses in the femoral neck region. A geometric adaptation that can improve ‘bone mechanical performance’ at critical fracture sites can help identify beneficial exercises and mobility patterns.

## Methods

### Data preparation

The data consisted of tomographic Magnetic Resonance (MR) images of the proximal femur of 111 participants - 91 female athletes (age: 24.7 ± 6.1 years) and 20 physically active women serving as a control group (age: 23.7 ± 3.8 years). The MR imaging protocol was based on axial T1-weighted gradient echo VIBE examination with (0.9 *mm* × 0.9 *mm*) in-plane pixel size and 1 *mm* slice thickness^1^. The study protocol was approved by the Ethics Committee of the Pirkanmaa Hospital District and each participant gave a written informed consent before the study. All methods implemented and used on the data for analysis in this study were in accordance with relevant guidelines and regulations. The athletes were categorised into mutually exclusive exercise loading groups by their characteristic loading patterns in their respective sports according to our standard procedure^1,25^: high impact (HI) group associated with maximal vertical jumps and high impacts (High jumpers and long jumpers, N = 19); odd impact (OI) group associated with rapid acceleration and deceleration as well as moderate to high impacts and bending forces from varying directions (Squash and soccer players, N = 19); high magnitude (HM) group associated with movements with coordinated high muscle force production at low rate (Powerlifters, N = 17), repetitive impact (RI) group associated with highly repetitive weight bearing impacts and bending forces (Endurance runners, N = 18) and repetitive non-impact (RNI) group associated with highly repetitive movements lacking ground impacts (Swimmers, N = 18). The anatomy of interest – proximal femur cortical geometry – was manually segmented from the MR image data of each participant. Apparently, the limited in-plane resolution of the native MR images compromises reliable inferences at locations with very thin cortices such as the femoral head and some regions of the femoral neck and the trochanters. However, it has been shown that MR-imaged cross-sections of the femoral neck are sufficiently precise and accurate for study purposes^26^. To extract the proximal femur cortical geometry, the image data of each participant was manually segmented by delineating periosteal and endosteal cortical surfaces. Thereafter the segmented geometry was converted into a volume mesh (10-node tetrahedral elements) for constructing a finite element (FE) model^24^. The nodes from the periosteal and endosteal surfaces of the volume mesh were extracted, along with the maximum and minimum principal strain values from the above-noted simulation results. The selected nodes were used to reconstruct the inner and outer triangular surface meshes of the cortical bone in MeshLab (Visual Computing Lab-ISTI-CNR, http://meshlab.sourceforge.net/). The nodes in the point cloud (*n* > 80,000) were first down-sampled to (*n* ≈ 25,000) using the Poisson disk sampling module^27^ and a surface reconstructed using the ball-pivoting module^28^. After checking for and cleaning any major errors (e.g. intersections, face flips, duplicates, holes) in the surfaces, they were remeshed to improve the quality (aspect ratio < 20) of the mesh in Avizo (FEI, Hillsboro, USA). The module implemented in this software attempts isotropic vertex placement based on Lloyd relaxation^29^. The final result of these preparation steps were the inner and outer surface meshes of the cortical bone. The image data included femoral anatomy from the femoral head to the proximal diaphysis of the femur below lesser trochanter. Thus, the open surfaces contained a single boundary at the lower extent of the data representing the distal extent of the femoral diaphysis. The feature vector at every node of the outer surface consisted of the cortical thickness and the maximum and minimum principal strains. Cortical thickness was calculated at the nodes of the outer surface and defined as the shortest distance to the inner surface.

### Planar parametrisation: Discrete Ricci-flow

**Figure 1:**
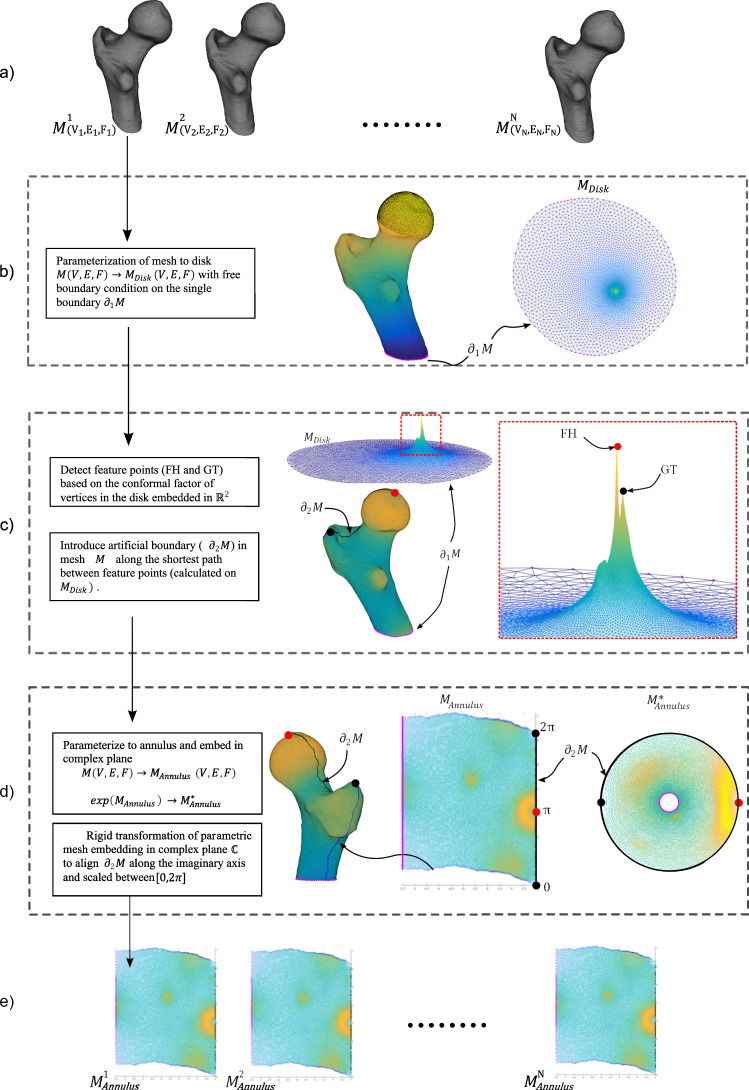
An illustrated description of the parametrising procedure developed for (**a**) proximal femur triangular surface meshes *M*_*N*_(*V*, *E*, *F*); where V = set of nodes; E = set of edges; F = set of faces (*N* = 111, in this study). (**b**) In the first parametrisation step, the surface is conformally mapped to its topological equivalent: disk. The single boundary (∂_1_*M*) at the distal end of the proximal femur (shaft) is mapped to the edge of the disk under a free boundary condition, where the metric on the boundary nodes is left unchanged (colour map: conformal factor). (**c**) The parametrised disk along with the conformal factor at the nodes as a height map. The femoral head (FH) and greater trochanter (GT) features are detected as the peaks (inset). The straight line between these features is used to introduce a second boundary (∂_2_*M*) by slitting the mesh along the line. (**d**) In the first parametrisation step, the surface is conformally mapped to its topological equivalent: annulus. The map is embedded in the complex plane by introducing a cut graph between the GT node and ∂_1_*M*. The embedded meshes are then transformed such that the ∂_2_*M* boundary lies on the imaginary axis scaled within [0, 2*π*]. An exponential map consequently results in the annulus. (**e**) Parametrised meshes in the a common coordinate frame. The boundary edges and feature points are colour coded consistently across all images.

To analyse and contrast the group-wise morphology of the proximal femurs, it was essential to establish correspondence between all individual surface meshes. The approach used in this work transforms the 3D surfaces into 2D (planar domain) using an angle preserving conformal method based on Ricci-flow. This approach leverages the methodological advantages of invariance towards rigid motion, scale and isometric deformations^21^. The residual deformations such as large non-isotropic deformations can then subsequently be accounted for in the 2D domain – which is a relatively simpler task. We present here a very brief description of the geometric basis for the implemented approach. For details, we refer readers to the textbook by Zeng & Gu where relevant references for the proofs can be found^30^. The discrete representation of the femur surfaces embedded in the Euclidean space ${{\mathbb{R}}}^{3}$ allows them to be treated as differentiable manifolds (2-manifold). Any surface in Euclidean space is a Riemannian surface with an inherent metric called Riemannian metric and a conformal structure. The Uniformisation theorem states that the Riemannian metric can be deformed to admit uniform curvature over the surface: +1, 0 or −1. Thus any closed Riemannian surface can be conformally mapped to one of these fundamental surface domains: unit sphere (+1), Euclidean plane (0) or hyperbolic plane (−1). The embedding in the relevant domain, referred to as parametrisation, reflects an angle preserving (conformal) transformation. Ricci-flow is a robust curvature flow method introduced by Hamilton^31^ that evolves the metric towards uniformisation as a heat diffusion process. It is a powerful method that provides the flexibility to design the final metric based on user-defined distribution of target Gaussian curvature. However, the total curvature is determined by the topology of the surface (*S*) according to the Gauss-Bonnet theorem: 2*πχ*(*S*). For an open orientable surface of genus (*g*) and number of boundaries (*b*), the Euler number is given by *χ*(*S*) = 2 − 2*g* − *b*. The genus of a closed surface can be intuitively thought of as the number of handles contained. The proximal femur data presented challenges that were addressed by tailoring specific solutions. The foremost challenge was the lack of anatomical correspondence at the distal boundary (femur diaphysis) of the surfaces across subjects because of different image field of views. This inconsistency of the boundary made it an unreliable reference for the subsequent correspondence detection. Thus appropriate references were derived from surface features through a two-step parametrisation procedure. The first step was devised to detect shape features to introduce a consistently defined reference feature for each mesh. The second step was devised to produce a mapping in a common coordinate space using the reference points for alignment (Fig. 1). The topology of the outer cortical surface (*χ* = 1; *g* = 0; *b* = 1) is conformally equivalent to a disk ${{\mathbb{R}}}^{2}$ (Fig. 1b). Ricci-flow mapping was performed by assigning zero target curvature to all interior points and leaving the boundary node metric unchanged (i.e. a free boundary condition). The resulting conformal factor distribution was used to investigate detectable surface features. Two prominent feature processes were identified from the disk parametrisation – the femoral head (FH) and the greater trochanter (GT). The two features can be seen as peaks whose representative centres were designated as feature points (Fig. 1c). These feature points were used consistently across all participants to introduce a stable reference in the form of an inter-feature geodesic. This was achieved by inserting a boundary (mesh slit) along the inter-feature geodesic path in the parametrised disk (i.e. straight line) and reflected in the native 3D surface. This modified surface (*χ* = 0; *g* = 0; *b* = 2) is conformally equivalent to a Euclidean annulus. The mapping was performed by assigning a target curvature of 0 to all nodes. A cut graph between the GT point and the distal boundary was calculated and the mesh slit along the edges. This enabled the fundamental domain of mapping to be embedded in the complex plane. Subsequently the embedded mesh was oriented so that the inter-feature boundary lay on the imaginary axis with the distal boundary parallel to it and incident on the negative real axis. Finally the mesh was resized such that the inter-feature boundary (on the imaginary axis) was scaled to [0, 2*π*] (Fig. 1d). The annulus was produced through an exponential map of the complex coordinates. The implemented MATLAB script accepts the 3D proximal femur surfaces as input and outputs the parametrised annulus with no user input required in between.

### Femur correspondence: planar domains

**Figure 2:**
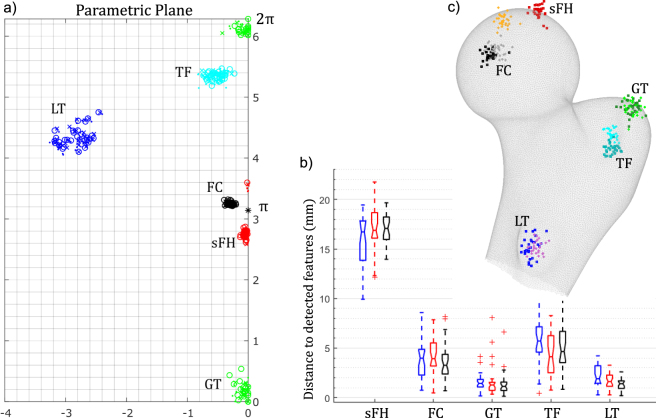
Assessment of the ability of the procedure to establish correspondence through expert annotations. 5 features were annotated by 3 experts on the surfaces of 30 subjects. The subjects were chosen randomly from the dataset of 111. The sites annotated were: superiormost point of femoral head (sFH), fovea capitis centre (FC), tip of greater trochanter (GT), trochanteric fossa centre (TF) and tip of lesser trochanter process(LT). (**a**) Clustering of features in the parametric plane: each surface was parametrised and the position of the annotated node plotted in the parametric plane. The sites are plotted in colour (sFH - red; FC - balck; GT - green; TF - cyan; LT - blue) and each annotator is indicated by a different marker (‘x’, ‘o’ and ‘.’). It should be noted that the boundary introduced along the geodesic between the two algorithmically detected features lies along imaginary axis (femoral head feature at *π* & greater trochanter feature at [0, 2*π*]). Thus, the GT and sFH annotations are reflected about this symmetry. (**b**) Box-plots of the distance between annotations and the associated feature, detected algorithmically, for each annotator (3 experts: each in blue, red and black). (**c**) A visual illustration of the distributions of the annotations (of one expert) and the detected features for 30 samples. Expert annotations are coloured in darker shades and detected features are coloured in lighter shades. The rendered surface of the femur is only for representation to convey a sense of location over the surface.

**Figure 3:**
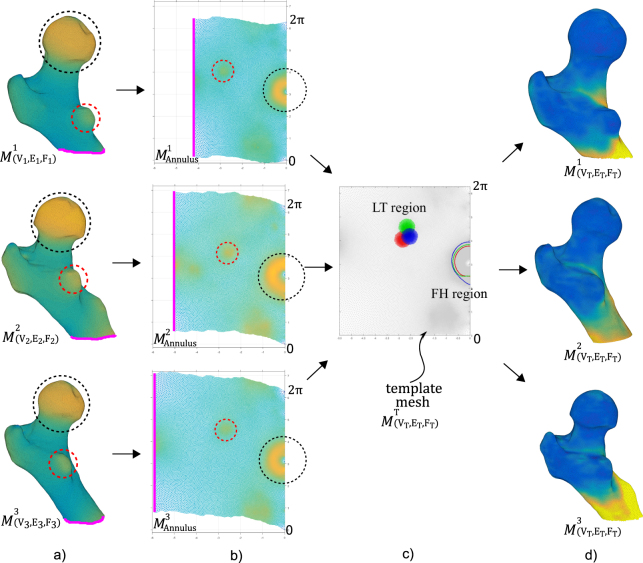
The correspondence procedure illustrated on 3 sample femur shapes from the dataset. (**a**) The three shapes chosen, were selected as they show clearly the differences in femoral neck lengths, shaft lengths and relative positions between the main feature processes - femoral head (FH), greater trochanter (GT) & lesser trochanter (LT). (**b**) The parametric meshes embedded in complex domain illustrate the positions of the FH (black circle) and LT (red circle). The consistent boundary of the mesh is aligned along the imaginary axis. (**c**) The canonical template mesh is matched to each of the parametrisations. The relative positions of the main features (LT and FH regions) in the common coordinate frame are illustrated in colour (red, blue and green for each of the 3 samples femurs). This misalignment is corrected locally on the template mesh through radial basis functions. (**d**) the resulting elastic registration of template mesh to each femoral instance produces an isotopological set of surfaces. The distribution of the cortical thickness values at the nodes are displayed as colour maps for illustrative purposes.

The result of the above-described parametrisation process produced 111 parametrised meshes embedded in a 2D complex coordinate frame. The embedded nodes benefit from the invariance accorded by the conformal method. That is, any node on the surface would retain its position in the parametric plane even if subjected to rigid, affine or isometric deformations in the native 3D domain. Consequently, the approach for establishing correspondence relied on matching a canonical template mesh (2D) to each of the individual 111 parametrized meshes. For each participant, the nodes of the template were designated with a natural representation^32^ which described their position with respect to the target mesh in barycentric coordinates. The 3D coordinates of the template nodes were calculated from the target nodes. This resulted in an elastic registration, where the template mesh conformed to the shape of every proximal femur surface in the dataset. The nodes of the template mesh were used as a dense set of corresponding feature locations across the 111 femur instances. If the variations in the proximal femoral anatomy only exhibited pure isometries then the template nodes would be aligned so that they represented correspondence. However, the shapes did exhibit differences that reflected anisotropic deformations. Thus, in the parametric plane, algorithmically detected features were mapped to clustered locations but were not precisely coincident. Figure 2 illustrates this for five distinct anatomic features^33^ that were independently annotated by three researchers experienced in femur anatomy, from 30 different proximal femora meshes randomly chosen from the dataset. Within each annotation cluster, their relative deviation from each other indicates the extent of anisotropy present in the sampled shapes. This deviation, and its contribution towards registration error, was reduced by locally deforming the parametrised template mesh to match the target mesh. In the present work, deformations of the template to correct anisotropic variations (Fig. 3) were based on two feature regions: femoral head and lesser trochanter (LT). Each region consisted of the feature point and the surrounding nodes, which acted as the feature support. The support nodes were defined as those within one standard deviation of the Gaussian surface centred on the feature point, that best fits the particular feature process. The feature processes were observed by mapping the conformal factor distribution over the planar parametrised mesh. The support nodes for each feature in the template were matched by deforming the nodes to occupy corresponding support region in the target parametrised mesh. The FH region was identified from the parametrised disk and the LT region was identified from the parametrised annulus. Smooth deformation of the template mesh was solved through Wendland based radial basis functions^34^, driven by the explicit deformations calculated within the two feature regions (FH and LT). To gauge the ability of the procedure in establishing correspondence, the mean distance between the annotations and their associated feature points (detected geometrically) were calculated for each annotator and illustrated as box plots (Fig. 2b). In order to visualize the 3D distribution of the features with respect to the annotations, the annotations of one expert for all 30 samples were mutually aligned using generalised Procrustes analysis. Subsequently, the corresponding transformations were applied to the associated feature points. This was used as a visual confirmation of the relative consistency in the distances between the features and the annotations (Fig. 2c). it should be noted that while the definitions of 4 landmark sites (FC, GT, TF and LT) coincide with their associated geometric feature points, FH and sFH are defined differently. FH represents the femoral head process as a geometric feature, while sFH is defined as the superiormost point on the head. This explains the large average distance between Fh and sFH. However, the plots indicate a consistency in their relative locations, displayed in the restricted spread in the box plots and the 3D distribution render.

The implemented script accepts the user defined template mesh and the stack of 111 parametrised target meshes. The output is a stack of morphed template meshes registered to each of the targets. The nodes of the morphed template meshes were attached with the natural representation with respect to their respective target meshes. The 3D coordinates at the nodes of the 111 morphed templates formed a set of 111 isotopological surfaces (i.e. same number of nodes and connectivity). The nodes were used as a dense set of correspondences established over the surface topologies across all individuals.

### Feature mapping and statistical analysis

After defining correspondence between the individual shapes, the average shape was calculated by generalised Procrustes analysis of the 3D coordinates attached to the nodes. The features of interest (thickness and principal strains) were calculated at the template node locations with respect to the target nodes of the individual mesh based on the natural representation. This resulted in feature maps for every participant, which were subsequently used for making statistical inferences. However making group-wise statistical tests at every node would introduce errors due to the large number of comparisons (*n* ≈ 25,000). Statistical parametric mapping (SPM) as implemented in the SurfStat package^35^ was used to correct for multiple comparisons (http://www.math.mcgill.ca/keith/surfstat/). The significance of the group term was tested by calculating the t-statistics, and subsequently random field theory based multiple comparison correction was performed. Patches were identified on the surface where the features of the loading group differed significantly from the controls. The identified patches represented a cluster of contiguous surface nodes that showed significance. In this study, a threshold of *p* < 0.005 was used for defining supra-threshold clusters and subsequently *p* < 0.05 as random field theory-correlated cluster threshold. Additionally, in order to account for the resolution limitation of the MRI dataset, regions with cortical thickness <1 *mm* were masked out in SurfStat. The spatial distribution of these clusters is visualised over the surface of the canonical femur to intuitively illustrate their extent. In this work, true anatomical correspondence was implicitly approximated through the parametrisation of representative geometric features. In the presence of anisotropic variations, the algorithm would likely systematically misregister the surfaces. To alleviate the effects of such registration errors the method recommended by Gee and Treece^36^ was implemented here by including shape modes as confounding factors. The first 5 shape modes which contain most (≈80%) of the variation were included in the SPM linear model^37,38^. The shape modes were calculated from vertex deformations, from their mean positions, following a generalised Procrustes alignment. As the scale was normalised in the procrustes analysis, the first shape mode did not reflect the scale and was thus included as a confounding factor. The linear model used for analysing the cortical thickness distribution involved the following variables – exercise loading group, body weight, shape and femur scale (size). Scale factor of each participant was defined relative to the average (canonical) shape which was transformed (Procrustes) to each shape in the isotopological dataset. The linear model variables for the principal strains (maximum and minimum) distribution were group, shape and individually estimated impact force. This impact force was obtained from the previous study^24^ and represented the applied force under which the respective FE models were simulated. The weight and height of each subject was used in calculating this force and thus, were excluded from the linear model.

### Data availability

The MATLAB code for the procedure developed in this study are available in the GitHub repository, Repository-link https://github.com/NathanielNarra/Femur-RicciFlow. The datasets generated during the current study and a select sample of 20 femur shapes from the dataset (for testing purposes) are also made available.

## Results

All parametrisation and registration processes were implemented in MATLAB. When the triangular surface meshes were treated for mesh quality, the parametrisation of a single femur mesh (≈25,000 nodes) took approximately 2 minutes on a desktop computer (Intel Xeon 2.4 GHz, RAM 54 GB). Bad triangle quality in the input mesh increased the convergence time of the evolving metric (≈4–5 mins).

**Figure 4:**
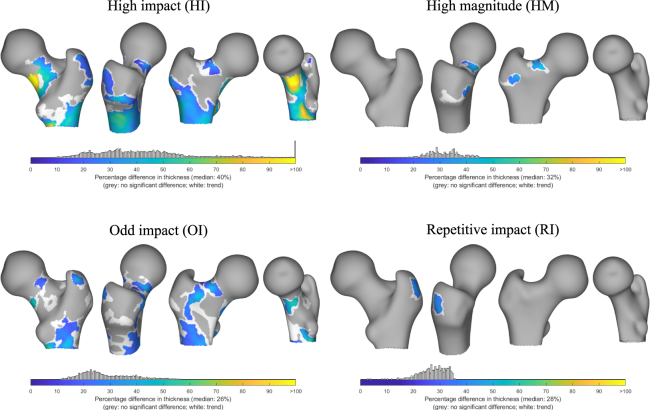
Regions of significant difference in cortical thickness (adjusted for weight, femur size and shape) illustrated on the surface of an average femur as colour patches. Colour maps in these patches represent mean percentage difference in cortical thickness between each exercise loading group and the control group (% higher than control). Their distribution within the patches is plotted as node counts above the colour bar. A relaxed multiple-comparison correction threshold (*p* < 0.025) for defining supra-threshold clusters (in white) was used purely to illustrate the trend in the distribution of these clusters; no formal inferences were made or discussed. The RNI group did not show any significant difference from controls, due to which it was omitted from this illustration.

The cortical thickness distribution in each loading group was contrasted with the control group, and statistical significances inferred by controlling for body weight, femur scale and shape (Fig. 4). The average femur shape of the entire dataset was used to illustrate the node clusters that differed statistically significantly from the control group. The percentage differences in cortical thickness at the nodes within these significant clusters were mapped in colour. In the HI group, the area covered by the clusters was the largest among all groups, indicating a greater extent of response in this group. Clusters were observed in the inferior and posterior regions of the femoral neck, with up to 90–110% thicker cortices. Significant response was also observed below the inter-trochanteric line in the metaphysis/proximal diaphysis regions, with mean differences in the identified clusters mostly in the range of 20–50% (median: 40%). In the OI group, large clusters were seen in the medial and posterior regions around lesser trochanter. Interestingly, a large cluster spanned the anterior aspect of the proximal femur; extending into the important superior region of the femoral neck. The mean percentage differences in the clusters were mostly in the range of 15–40% (median: 26%). The HM group showed a relatively minor cluster extent in the superior and supero-anterior regions of the femoral neck; with the mean percentage difference in the cluster within 25–40% (median: 32%). The RI group showed a small region of significant clusters with a median 28% difference in cortical thickness on the lateral side of the greater trochanteric process. The RNI group did not show significant differences in the cortical distribution from the control group.

**Figure 5:**
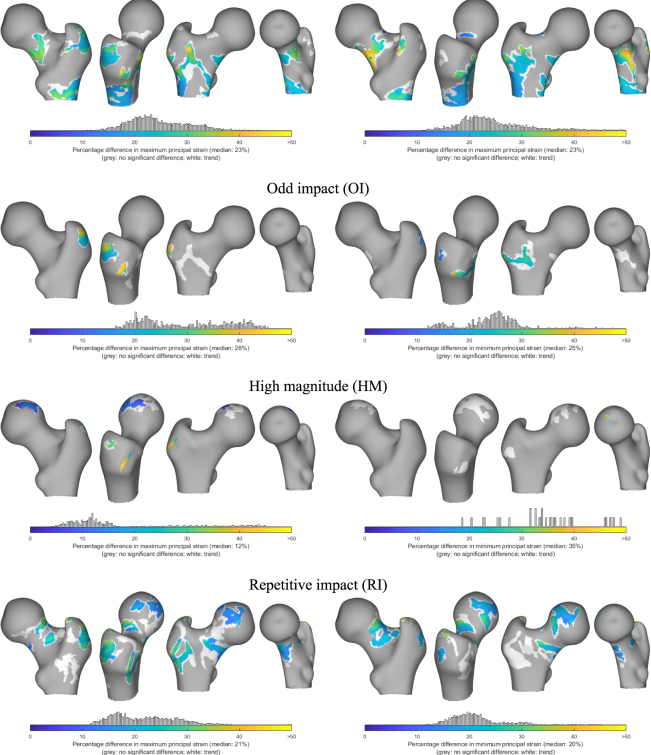
Regions of significant difference in maximum and minimum principal strains at the surface nodes (adjusted for individual impact force and shape) illustrated on the surface of an average femur as colour patches. Colour maps in these patches represent mean percentage difference in the principal strains between each exercise loading group and the control group (% lower than control). Their distribution within the patches is plotted as node counts above the colour bar. A relaxed multi-comparison correction threshold (*p* < 0.025) for defining supra-threshold clusters (in white) was used purely to illustrate the trend in the distribution of these clusters; no formal inferences were made or discussed. The RNI group did not show any significant difference from controls, due to which it was omitted from this illustration.

Surface maximum and minimum principal strain distribution revealed differences in response regions when exercise loading groups were contrasted with the controls (Fig. 5). Statistical significances were inferred by controlling for estimated individual impact force during a sideways fall. The HI group showed large cluster extents in the inferior and posterior regions of the femoral neck. Clusters were also observed in the anterior region roughly extending distally from the inter-trochanteric line. In the OI group a large cluster was found spanning the anterior aspect of the inter-trochanteric line. The RI group showed large clusters nearly all around the femoral neck region except a small span at the supero-anterior region. Other clusters in the RI group were also found in the lateral region of the greater trochanter and superior region of the femoral head. In the HM group, only a tiny cluster was found while the RNI group showed no significantly differing clusters from the control group.

**Figure 6:**
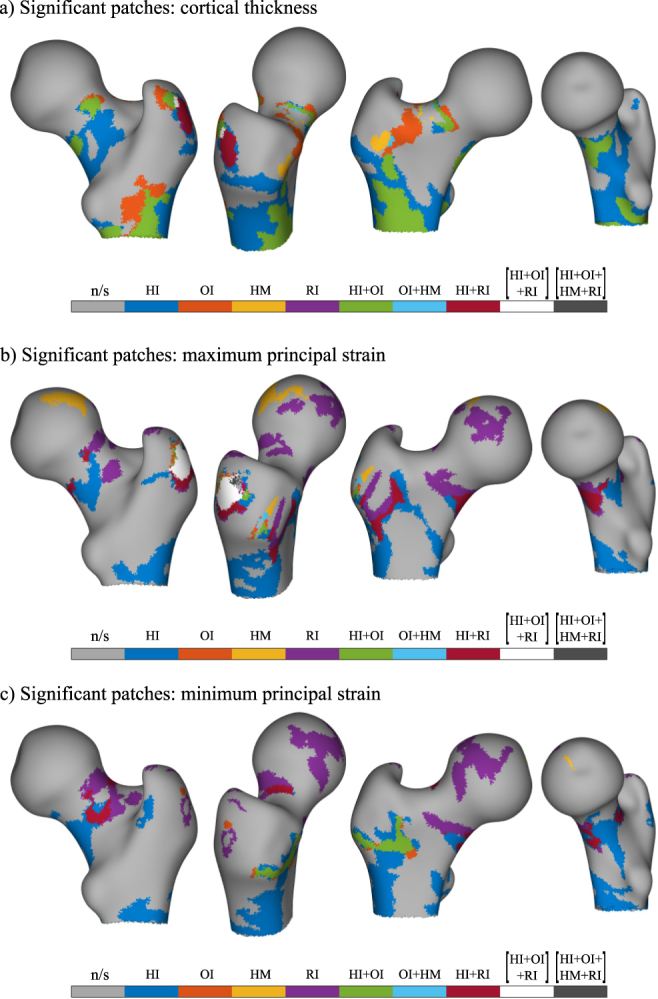
The significant clusters from the loading groups are combined into a single map. The regions are colour coded according to the specific or combination of loading groups responsible (n/s: no significance). The combination regions are where multiple loading groups (as indicated) show significant differences from controls. Compiled from the results for (**a**) cortical thickness (**b**) maximum principal strain (**c**) minimum principal strain.

The group-wise clusters were used to construct a single composite surface visualisation for cortical thickness (Fig. 6a), maximum principal strain (Fig. 6b) and minimum principal strain (Fig. 6c). Here, unique regions were identified that showed significant response to specific loading regimes. Moreover, regions that might respond to combinations of different loading types were also identified.

## Discussion

In large sample analyses of the proximal femur geometry, the co-registration of multiple femur instances is an essential pre-processing step. The task of establishing correspondence is commonly done by first removing rigid motion to normalise the global pose of the femur shapes. Subsequently non-rigid registration is performed, relying typically on iterative closest point and b-splines to perform non-rigid registration. The external phenotype of the femur is relatively simple (as a geometric topology) and largely similar within population. However, distinct anatomical features that can be reliably detected are sparse in number. Some studies have supplemented the count with derived landmarks and have created procedures that perform the identifications autonomously^39^. A majority of these features are located in the proximal and distal epiphysis of the femur. The femur dataset studied in this work is not only incomplete representing the proximal part only but also inconsistent in its anatomical extent, which made the registration process challenging. Thus, an elegant solution was sought to manage these challenges and in turn to develop a tool for proximal femur registration.

This study presents an application of Ricci-flow based conformal surface parametrisation for analysing the proximal femur morphology. The implemented procedural chain defines correspondence between the surface meshes by using only three consistently detectable anatomic features (automated landmarks). In its current form, the algorithm relies on the presence of the complete epiphysis to enable the detection of the femoral head and greater trochanter processes. Inconsistencies in the femur anatomy distal to the inter-trochanteric line are accommodated. Thus the method was designed to establish correspondence between surfaces with varying femur shaft lengths. The robustness of Ricci-flow based conformal mapping method was leveraged to establish isometry invariant global correspondence through a dimension reduction approach (3D $\mapsto$ 2D). This could be observed in Fig. 3 where the three sample femurs illustrate differences in femoral neck length, shaft length and lesser trochanter position. It can be seen that the protrusion near the lesser trochanter (Fig. 3a, mesh: ${M}_{({V}_{2},{E}_{2},{F}_{2})}^{2})$ and the varying shaft lengths in all three did not skew the registration process. The residual variations in the shape that are not isometric in nature can manifest as differences in the relative distances between the features. This can be observed by the plotted lesser trochanter regions and the head-neck contour (Fig. 3c). Residual variations were locally handled in the 2D planar domain using simple 2D deformation methods, specifically using radial basis functions.

While the procedure developed in this work is entirely tuned towards the analysis of the proximal femur, the Ricci flow method provides flexibility to handle any topology. By perceiving the surface data as a geometric manifold, their topological invariance can be used to compute conformal maps. Working within the constraints of the Gauss-Bonnet theorem, consequently any number of parametrisations can be computed to facilitate feature detection. Different parametrisations result in different conformal factors distributions that can be used as additional parameters in detecting surface features. In this work, the femur was parametrised in two different ways to detect three anatomic features, represented in terms of their geometric shape. Disk parametrisation was used to detect the greater trochanter and femoral head, while the annulus parametrisation was used to detect the lesser trochanter. Though not used in this work, multiple such parametrisations can be used to further decrease area distortion related registration errors^32^. Further avenues for improvements that will be explored in the future include using features like the trochanteric fossa, fovea capitis and the greater trochanteric process. Geodesics between features (e.g. inter-trochanteric line) can be used to improve co-registration between shapes in the inter-feature regions to estimate the closest anatomical correspondence.

The developed method was applied to investigate morphological adaptations in the proximal femur in response to long-term exercise loading. The 3D distribution of the cortical thickness was contrasted between five exclusive exercise loading groups and controls. Analysing its distribution over the surface helped localise the regions, in terms of coloured patches of surface node clusters, where the response was significant in comparison to controls. Thus, the present study complements the inferences drawn from previous studies with this data^1,2,24,40^. The HI and OI groups showed large clusters, while the HM and RI groups showed small clusters. The RNI group showed no significant response. In the HI group the clusters were broadly in the inferior, anterior and posterior regions of the femoral neck; at and below the lateral aspect of the greater trochanter and in the proximal diaphysis below the inter trochanteric line. In the OI group, smaller patches were observed in the femoral neck region and the proximal diaphysis, the regions mostly coincident with those of HI group. The HM group showed much smaller clusters but they were located in the superior and supero-anterior sector of the femoral neck. The RI group had a single cluster located at the lateral side of the greater trochanter process. In terms of cortical thickness, the results clearly indicate that the HI and OI groups demonstrate spatially diverse response. The composite map in Fig. 6a, maps identified clusters to the specific exercise loading group or their combinations.

Similar analysis of the maximum and minimum principal strains at the surface nodes extracted from a simulation study revealed large response regions in the HI and RI groups. Smaller regions were observed in the OI and HM groups, while the RNI group did not differ from the controls. The composite images in Fig. 6b,c shows that the response in the RI group is spatially distributed around most of the femoral neck. In the HI group, the response in the femoral neck is predominantly in the inferior and posterior regions. Interestingly, the OI and HM group showed very little response. When comparing with the results of octant analysis of von Mises stresses performed by Abe *et al*.^24^, the present results suggest that the OI group do not differ from controls in terms of principal strain. The discrepancy in the OI group between the present and earlier study may be due to the fact that only the surface strains were studied here while the previous study analyses stresses through volume aggregates within octant sectors. Thus in the future a more comprehensive study will be conducted for volume averaged stress and strain maps. Nevertheless, the surface stain maps illustrate an apparent increase in robustness due to repetitive loading activity (RI group), seemingly achieved without an associated increase in cortical thickness. This is in line with the results reported in the study by Abe *et al*.^24^, and based on the cross-sectional shape differences for this group reported by Narra *et al*.^2^, it likely points to the role of curvature of the cortical shell in the improved performance. Thus, the procedure of mapping FE simulation results, enhances the ability to explore and localise significant patterns in the distribution of mechanically relevant features.

The method developed and the case study have limitations that ultimately temper the derived statistical inferences. The relatively low in-plane resolution of the MR images, while shown to be sufficient for the analysis of the entire femoral neck cross-section^26^, precludes drawing conclusions in regions with very thin cortices locally (e.g. femoral head and some femoral neck regions). In addition, the highly adaptive trabecular bone which plays a significant role in buttressing the thin cortical shells in the proximal femur were not included in this analysis. Thus, the results should not be interpreted as an exhaustive study of the total adaptive response due to each of the loading types. The analyses should be read within the context of purely geometric changes in the cortical shell due to long-term habitual exercise. In the registration task, manual annotations on a sample subset illustrated the insufficiency of the conformal method in accommodating anisotropic shape differences. While the isometric invariance of the method mapped all annotations into clusters, the spread of these clusters indicates the anisometric deformations between shapes (Fig. 2a). These deformations were only explicitly handled at the three algorithmically detected features (LT, GT and FH), which may be insufficient to establish precise correspondence in the inter-feature regions. Moreover, these three features do not necessarily represent anatomical features, as their detection was performed in terms of the surface geometry. Residual errors in terms of true anatomical homology probably persist (Fig. 2b). Sliding semilandmarks-based^41^ procedures can be used to improve correspondence in the vicinity of identifiable features. They typically require manual annotations of specific sites, which can potentially be extended to work with features detected on the parametrised surface. Alternatively, best-fit based local searches can be implemented to account for anisometries in the inter-feature regions to increase the accuracy in correspondence. Similarity metrics such as mean curvature and conformal factor (i.e. area distortion) can be used^32^. It should be noted that there are faster tools based on ICP that can be used to establish acceptably imprecise correspondence. However, they introduce limited arbitrariness due to the reliance on the initial pose estimation and are susceptible to noise in the surface. The conformal map approach to the issue of shape matching has many appealing qualities, foremost among which are: (1) consistency in the treatment of surfaces regardless of initialization, surface noise and isometric deformations; (2) utility in registering incomplete anatomies without a need for either fine tuning initial pose or enforcing constraints. We demonstrated an unexplored application of conformal mapping to the proximal femur where distinct features are few. Its potential can be realised by employing more sophisticated 2D matching processes of the parametrised meshes (2D registration) to improve correspondence in the inter-feature regions. Thus, the present method can be used to address specific challenges posed by data collection such as incomplete surface data and anatomies with sparse features.
